# Boosting Bioluminescence Neuroimaging: An Optimized Protocol for Brain Studies

**DOI:** 10.1371/journal.pone.0055662

**Published:** 2013-02-06

**Authors:** Markus Aswendt, Joanna Adamczak, Sebastien Couillard-Despres, Mathias Hoehn

**Affiliations:** 1 In-Vivo-NMR Laboratory, Max-Planck-Institute for Neurological Research, Cologne, Germany; 2 Institute of Molecular Regenerative Medicine, Paracelsus Medical University, Salzburg, Austria; 3 Spinal Cord Injury and Tissue Regeneration Center Salzburg (SCI-TReCS), Salzburg, Austria; University G. D'Annunzio, Italy

## Abstract

Bioluminescence imaging is widely used for optical cell tracking approaches. However, reliable and quantitative bioluminescence of transplanted cells in the brain is highly challenging. In this study we established a new bioluminescence imaging protocol dedicated for neuroimaging, which increases sensitivity especially for noninvasive tracking of brain cell grafts. Different D-Luciferin concentrations (15, 150, 300 and 750 mg/kg), injection routes (iv, ip, sc), types of anesthesia (Isoflurane, Ketamine/Xylazine, Pentobarbital) and timing of injection were compared using DCX-Luc transgenic mice for brain specific bioluminescence. Luciferase kinetics was quantitatively evaluated for maximal photon emission, total photon emission and time-to-peak. Photon emission followed a D-Luciferin dose-dependent relation without saturation, but with delay in time-to-peak increasing for increasing concentrations. The comparison of intravenous, subcutaneous and intraperitoneal substrate injection reflects expected pharmacokinetics with fastest and highest photon emission for intravenous administration. Ketamine/Xylazine and Pentobarbital anesthesia showed no significant beneficial effect on maximal photon emission. However, a strong difference in outcome was observed by injecting the substrate pre Isoflurane anesthesia. This protocol optimization for brain specific bioluminescence imaging comprises injection of 300 mg/kg D-Luciferin pre Isoflurane anesthesia as an efficient and stable method with a signal gain of approx. 200% (compared to 150 mg/kg post Isoflurane). Gain in sensitivity by the novel imaging protocol was quantitatively assessed by signal-to-noise calculations of luciferase-expressing neural stem cells grafted into mouse brains (transplantation of 3,000–300,000 cells). The optimized imaging protocol lowered the detection limit from 6,000 to 3,000 cells by a gain in signal-to-noise ratio.

## Introduction

Noninvasive imaging has gained high interest for cell tracking approaches in preclinical studies of stem cell transplantation. Bioluminescence imaging (BLI) is one of the methods to overcome the restrictions of conventional invasive histological evaluations of stem cell fate and localization. Bioluminescence (BL) is based on the oxidation of the substrate D-Luciferin in the presence of oxygen and ATP by the enzyme luciferase, which was first isolated from the firefly *Photinus pyralis* [1]. Cell localization is achieved by the specific light signal generated only by genetically modified cells expressing a luciferase gene. In case of the firefly luciferase the enzyme reaction is ATP-dependent and therefore serves also as an indicator for cell viability. In addition, gene expression is quantifiable based on the linear relationship between substrate and photon emission.

In vivo BLI is challenged by tissue related light absorbance from hemoglobin and melanin. The high blood to brain tissue ratio is particularly unfavorable for most luciferase imaging since their wavelength-windows of maximal emission overlap with the maximal absorption of hemoglobin. The photon transmission in living brain tissue is limited substantially by the multilayer anatomical barriers (skin, bone, meninges, brain tissue). Furthermore, the blood brain-barrier reduces diffusion of the luciferase substrate or even selectively transports the substrate back to the blood system [2].

For a maximized sensitivity, the technical set-up especially with an up-to-date CCD camera system is a prerequisite. At the cellular level, the photon emission per cell can be maximized by using a strong promoter and single cell cloning approaches to identify high-expression clones, which enable the detection of individual subcutaneous tumor cells in vivo [3]. On the other side, physiological parameters during in vivo imaging such as the type of anesthesia [4] and the route of substrate delivery [5], [6] influence decisively BL signal outcome and experimental stability. D-Luciferin biodistribution and cell uptake kinetic studies have revealed a limitation of light emission due to restricted substrate availability, particularly in the brain [7]. Nevertheless, the general imaging protocol seems to vary substantially between studies.

Own survey on recent BLI literature indicated that many labs use an ip injected luciferin dose of 150 mg/kg under Isoflurane – a condition which we call “standard protocol” in the following investigation. The aim of our present study was to establish an optimized protocol for BLI sensitivity and reproducibility, dedicated for imaging investigations on the rodent brain. For this purpose, we assessed the following parameters influencing the BLI signal: 1) substrate concentration, 2) substrate injection route, and 3) type of anesthesia (Isoflurane, ketamine/xylazine, Pentobarbital) and its timing relative to substrate application. The effect of the above listed parameters on maximal photon emission (PE_max_), on total photon emission (represented by area-under-curve, AUC), and on in- and efflux kinetics of D-Luciferin (time-to-peak) was investigated. Brain-specific BLI was obtained with DCX-Luc transgenic mice expressing firefly luciferase specifically under control of the doublecortin (DCX) promoter, which is active in migrating neuroblasts and early-stage differentiated neurons. This mouse model is proven to serve as a suitable tool to follow neurogenesis in the brain [8]. Based on the evaluation of the different protocols with the transgenic mouse line, we tested the optimized protocol on nude mice, which received implants of distinct amounts of neural stem cells (NSCs) expressing the firefly luciferase. This in vivo dilution series of grafted cells served to validate our optimized protocol for maximized sensitivity for brain imaging applications.

## Materials and Methods

### 1. Animals and Ethics Statement

Heterozygous DCX-Luc mice (n = 4) with C57/Bl6 albino background (B6(Cg)-Tyrc-2J/J) were used for optimization of the brain BLI protocol. Experiments were carried out with 4–6 months old mice. For BLI optimization, each DCX-Luc mouse underwent 12 times BLI measurements including D-Luciferin injections and anesthesia. In general, the time delay between two experiments were 2 days or more. Cell transplantations were performed with 12 male Nu/Nu mice (Janvier, Saint Berthevin Cedex, France). These mice were measured with two different BLI protocols with a time delay of 6 h or more. All animal experiments were conducted according to the guidelines laid out in the German Animal Welfare Act, in accordance with the European Council Directive 2010/63/EU, and were approved by the local authorities (Landesamt für Natur, Umwelt und Verbraucherschutz North Rhine-Westphalia, reference number 84-02.04.2011.A123). Animals were housed in individually ventilated cages under 12 h light/12 h darkness cycle with access to water and food *ad libitum*.

### 2. Cell Culture

The generation of neural progenitor cells (NPCs) from murine embryonic stem (mES) cells was achieved by adaption of previously described protocols [9]. The mES cell line D3 [10] (a generous gift of Dr. Tomo Saric, Institute of Neurophysiology, University at Cologne, Germany) was cultured on mitotically inactivated mouse fibroblast cells as previously described [11]. Embryoid body (EB) formation was induced in suspension for 4 days in DMEM supplemented with 15% FCS (Gibco, Darmstadt, Germany), 1× non-essential amino acids (PAA, Pashing, Austria), 2 mM L-glutamine (PAA), 1% penicillin/streptomycin (PAA). EBs were converted into neurosphere-like aggregates by culturing in N2/B27 medium consisting of DMEM/F12 (Gibco), Neurobasal (Gibco), 1× B27 (Gibco), 1× N2 (Gibco) and 2 mM L-glutamine (PAA), changing medium every 3–4 days on Poly-HEMA (Sigma-Aldrich, Schnelldorf, Germany) coated flasks. After 10–14 days, neurospheres were plated on flasks coated with 0.2% gelatin (Sigma-Aldrich). 24 hours later, medium was changed to N2Euro medium consisting of Euromed (Biozol, Eiching, Germany), 10 ng/ml EGF (Peprotech, Hamburg, Germany), 10 ng/ml b-FGF (Peprotech), 50 µg/ml BSA (PAA), 25 µg/ml Insulin (Sigma-Aldrich) and 1× N2 (Gibco). The N2Euro medium promoted proliferation of EGF and b-FGF responsive stem cells, while non-NSCs were removed by culturing and passaging with Accutase (PAA) for 20 days until the final D3WT_N2Euro cell line was established. Cells were cultured under humidified 5% CO_2_ and 95% air.

### 3. Cloning of Luc2 Retroviral Plasmid and Generation of Transgenic NPCs

The retroviral plasmid pBabe-Luc2-T2A-copGFP-SV40P-Neo consisted of the codon optimized firefly luciferase Luc2, the “self-cleaving” 2A-like peptide sequences T2A for efficient multicistronic reporter expression, the superbright fluorescent protein copGFP from the copepod *Pontenilla plumata* (excitation/emission maximum = 482/502 nm) [12] and the antibiotic resistence gene neomycin (Neo) for clone selection. Retroviral packaging was done according to established protocols with transfection of the packaging Plat-E cells, which express the gag-pol and ecotropic envelope proteins [13]. The plasmid was cloned by amplifying recombinant DNA by PCR using specific primers bearing appropriate restriction sites in 2 steps: 1) Luc2 was cloned from pGL4.14 (Promega, Madison, USA) into pCDH-EF1-MCS-T2A-copGFP (Biocat, Heidelberg, Germany) with the following primer pair Luc2-*BamHI*-for (AAGGGAAA*GGATCC*GCCACCATGGAAGATATGCCAAAAACATTAAG) and Luc2-*NotI*-rev (AAATTT*GCGGCCGC*CACGGCGATCTTGC), 2) the resulting Luc2-T2A-copGFP fragment was cloned into pBabe-Neo-GIT (a generous gift of Prof. Andreas Jacobs, European Institute of Molecular Imaging, Münster, Germany) by restriction with BamHI and NotI. Successful cloning was verified by restriction analysis and sequencing.

### 4. Ecotrope Transduction and Selection of Stable Transgenic NSCs

Retroviral packaging of Plat-E cells was done according to manufactureŕs protocol with small modifications (Cell Biolabs, San Diego, USA). Briefly, Plat-E cells were plated 24 h before the transfection at 1×10^5^/cm^2^ in 0.25 ml/cm^2^ DMEM FCS (without antibiotics). The transfection mix consisted of 0.2 µg/cm^2^ plasmid DNA, 20 µl/cm^2^ Optimem (Gibco) plus 0.2 µl/cm^2^ Lipofectamine Plus Reagent, and 0.25 µl/cm^2^ Lipofectamine (Gibco) and was added dropwise to the cells covered with 0.1 ml/cm^2^ Optimem containing Chloroquine (final conc.: 25 µM) and incubated for 24 h. After virus collection 48 h post transfection, the virus was used for retroviral infection of proliferating D3WT_N2Euro cells (non-confluent culture in 24-well plate), by adding 200 µl Optimem containing 7.5 ng/ml Polybrene and 200 µl virus-supernatant for 24 h. In a first step of transgenic cell selection, 500 µg/ml G-418 was added to the media. In a second step, cells were FACS-sorted (FACSAria III, BD Biosciences, San Jos, USA) according to the copGFP expression – performed to assure Luc2 expression in an equimolar range. Sustained cell viability was confirmed by PrestoBlue assay according to the manufactureŕs protocol (Invitrogen, Darmstadt, Germany) and measured with a microplate reader (Mithras LB940, Berthold, Bad Wildbad, Germany). In the following, we will refer to the transgenic NSCs as NSC^Luc2+^ and to non-transduced NSCs as NSC^WT^.

### 5. Cell Transplantations

Nude mice were anesthetized with Isoflurane in O_2_:N_2_O (30∶70%), and 4 mg/kg Carprofen (Pfizer, Berlin, Germany) was used for analgesia. During surgery, mice remained fixed in a stereotactic frame (Stoelting, Dublin, Ireland). The skull was exposed by a small incision and one hole was drilled at the following coordinates relative to bregma: AP +0.5; L +2.0; DV −3.0 using a stereotactical instrument (Stoelting). The homogeneous cell suspensions were kept on ice during surgery and were subsequently injected into the brain through a Hamilton syringe (26G needle) using a micropump system with flow rates of 1.500 nl/min (withdrawal) and 500 nl/min (injection). Cell amounts were adjusted to 3×10^3^, 6×10^3^, 3×10^4^ and 3×10^5^ cells (n = 3 for each cell number) and injected in a volume of 2.0 µl after leaving the needle in place for 5 min. The wound was closed with a non-colored suture (Resorba, Nürnburg, Germany), and BLI was applied 24 h later.

### 6. In vitro and in vivo Imaging Set-up

D-Luciferin (Synchem, Felsberg, Germany) solutions were prepared by dilution in phosphate buffered saline (PBS) to obtain 3, 20, 50 and 100 mg/ml stock solutions, which were filtered sterile prior to use and stored at -20°C. In vitro sensitivity was evaluated with NSC*^Lu2+^* and NSC*^WT^* plated in different cell densities of 3×10^3^, 6×10^3^, 3×10^4^ and 3×10^5^ cells in black 96-wells (Corning, Tewksbury, USA), in four wells per condition. D-Luciferin was added in excess (0.3 mg/ml, ≈1 mM according to [1]) and the plate was subsequently measured for BLI recording data on the Photon Imager (Biospace Lab, Paris, France) for 1 min.

Before the first BLI recording, animals were shaved under Isoflurane anesthesia on the head region and on the back of similar size using a conventional hair shaver (Typ 1556, ermila, Unterkirnach, Germany). Shaving was repeated when necessary according to visual inspection. Animals were injected intraperitoneally (ip) using a 1 ml syringe with 30 gauge needle. For intravenous (iv) injections, a catheter with 30 gauge needle was placed into the tail vein, flushed with saline and 1 units/ml heparin (Braun, Melsungen, Germany). Injection volume for different D-Luciferin concentrations (15–750 mg/kg) was kept constant (approx. 200 µl) by using the appropriate stock concentration. Anesthesia was induced either by 2% Isoflurane (Iso) in 100% O_2_, or subcutaneous (sc) injection of 60 mg/kg Pentobarbital (Pento), or ip injection of 100/10 mg/kg Ketamine/Xylazine (Ket/Xyl). The impact of anesthesia on photon emission was investigated in 4 DCX-Luc mice by comparing the standard procedure −150 mg/kg post Isoflurane (post-Iso) to substrate administration pre Isoflurane (pre-Iso) and injection of 150 m/kg after Pentobarbital or Ketamine/Xylazine anesthesia. In order to induce rapid Isoflurane anesthesia for the pre-Iso condition, an initial dose of 4% Isoflurane in 100% O_2_ was used. The time lag between substrate injection and acquisition was recorded during each BLI experiment and used for time-line correction (acquisition time = 30 min) in order to facilitate precise data evaluation. The time lag ranged from 30 s (e.g. ip injections) to 3 min (e.g. iv injections and pre-Iso condition).

### 7. Data Analysis and Statistics

For in vitro analysis, photon emission per NSC*^Luc2+^* was calculated from a dilution series by dividing the photons/s/cm^2^/sr per well by the cell amount and averaging of 4 individual experiments.

In vivo BLI data was analyzed using the M3software (Biospace) with size-constant regions of interest (ROIs) over the brain and the back of the animal, manually drawn and based on anatomical landmarks (ears, eyes) on equally scaled BL images. Further calculations, plotting and statistical analysis were done with Veusz (GPL, Jeremy Sanders) and Origin 8G (OriginLab Corporation, Northampton, USA) according to previously described methods [14]. Dynamic time curves were obtained with 5 and 60 s temporal resolution for two regions of interest (ROIs) for each animal and experiment. The brain-specific signal was corrected for unspecific signal taken from a ROI on the skin of the animal’s back. The 5 s and 60 s data from the ROI on the animal’s back was subtracted from the 5 s and 60 s brain data. This correction reduced the inter-individual differences from endogenous variations in DCX-expression and exogenously administered D-Luciferin (see Supplementary Fig. 1). The maximum photon emission (PE_max_) was determined from the acquisition of the signal-time curve, recorded with 5 s temporal resolution and by calculating the 95^th^ percentile. The area-under-curve (AUC) value was calculated by numerical integration for comparing the total photon yield from the same acquisition. The maximum value in the 60 s resolution data was used for defining the time-to-peak value. Furthermore, the slope of the signal increase during the initial 5 min was determined for every acquisition by linear fitting of the data points. Signal-to-noise ratio (SNR) was calculated by dividing mean photon flux (ph/s/cm^2^/sr) by the standard deviation of the noise. For accurate calculation of the noise, the mean of 3 randomly chosen ROIs outside the mouse was calculated.

**Figure 1:**
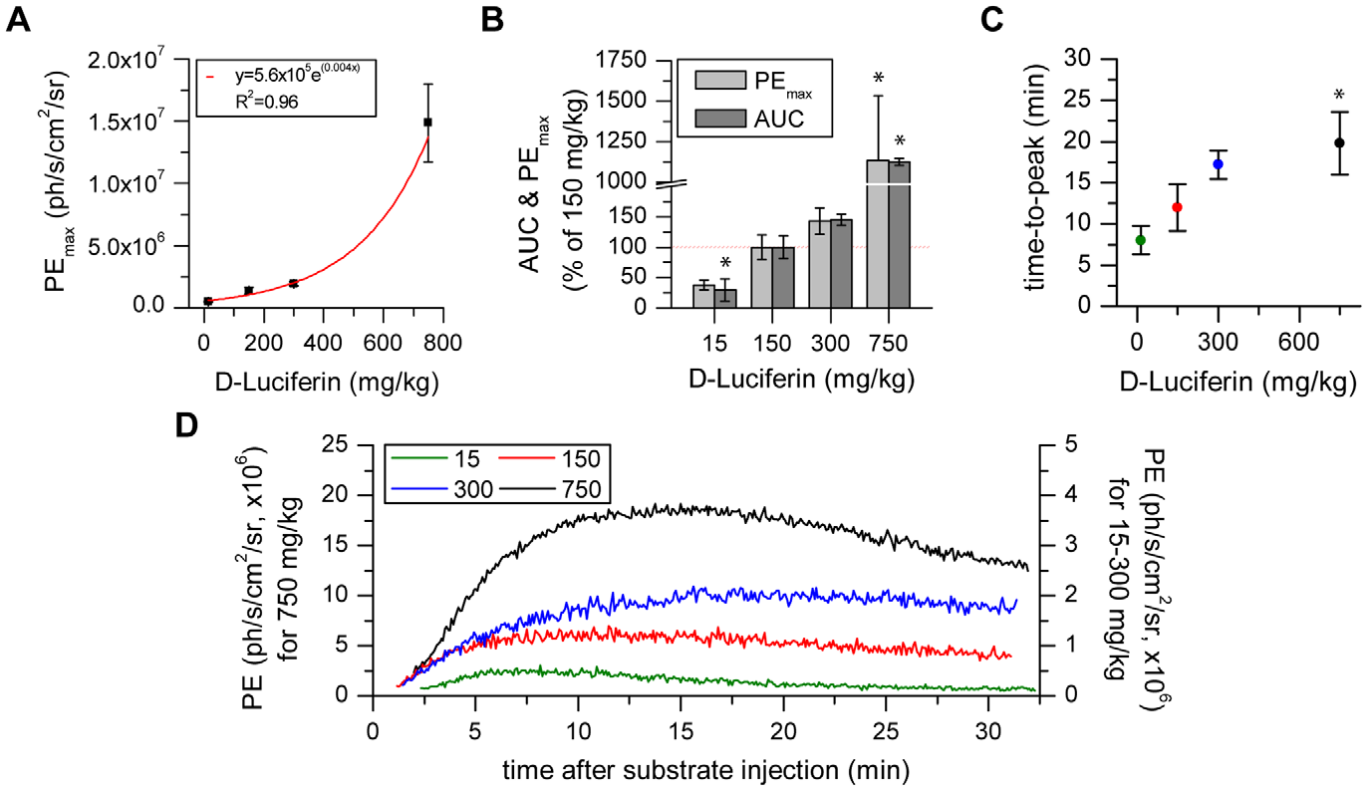
Substrate concentration modulates luciferase activity. a, b) The PE_max_ and AUC values increase exponentially with the D-Luciferin concentration. c) The time for maximal luciferase activity is dependent on the substrate concentration. d) The slope of initial photon emission kinetics is concentration dependent. (* statistically significant difference with p≤0.05 to standard protocol 150 mg/kg post-Iso in post hoc comparison with Sidak correction).

All data are represented as mean ± SD. Unpaired t test was used to compare means of 2 groups for in vitro experiments. In vivo BLI data was analysed using a repeated measures ANOVA for each individual test regime (concentration, injection route, anesthesia, time of injection). Post hoc comparisons were corrected for multiple comparisons using Sidak correction. A p-value smaller than 0.05 was considered to be significant.

## Results

### 1. Substrate Concentration Dependent Increase in Photon Flux

The impact of substrate concentration on photon emission was investigated by injecting 15, 150, 300 or 750 mg/kg D-Luciferin ip into DCX-Luc mice (n = 4) after 2% Isoflurane anesthesia, and subsequent BLI recording for 30 min. Substrate concentration had a significant effect on PE_max_ (F(1.006, 3.017) = 72.862 p = 0.003), AUC (F(1.005, 3.016) = 66.988) p = 0.004) and time-to-peak (F(3, 9) = 28.693, p<0.001). The PE_max_ signal exponentially increased with substrate concentration in every animal, as well as the total quantum yield represented by the AUC (Fig. 1a). A 5-times standard concentration resulted in approx. 10-fold PEmax/AUC value, together with increased error (standard deviation). This strong signal enhancement was accompanied by higher slope values in the initial inflow phase following also the exponential relationship (1.1×10^5^–2.9×10^6^) and a prolonged time-to-peak for high substrate concentrations (Fig. 1b, c).

### 2. Influence of Substrate Injection Route

**Figure 2:**
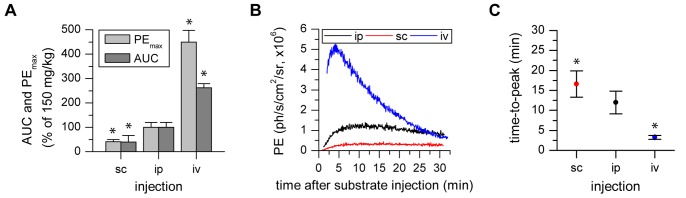
The photon flux maximum and timing are dependent on the route of substrate administration. a, b) The PE_max_ and AUC increase corresponding to the physiologically expected biodistribution for sc, ip and iv substrate administration, reflected by the characteristic time activity curves. c) Maximal photon flux is reached at minimal time for iv injections followed by ip and sc (* statistically significant difference with p≤0.05 to standard protocol 150 mg/kg post-Iso in post hoc comparison with Sidak correction).

The impact of substrate injection routes was evaluated by injecting 150 mg/kg D-Luciferin ip, sc, or iv followed by acquisition for 30 min in DCX-Luc mice (n = 4). Injection route had a significant effect on PE_max_ (F(2, 6) = 75.048 p<0.001), AUC (F(2, 6) = 126.935 p<0.001) and on time-to-peak (F(2, 6) = 71.148 p<0.001). According to the general model of drug absorption and resorption [15], the PE_max_ for sc reached approx. 40% of the ip experiment, whereas iv resulted in approx. 450% PE_max_. The AUC values showed a similar behavior with a minor effect for iv - approx. 260% (Fig. 2a). The absorption kinetics was also reflected by the BLI dynamic time curves with a smaller slope for sc vs. ip (6.1×10^4^±2.2×10^3^ vs. 2.2×10^5^±8.6×10^3^). The absorption-independent iv injection reached with 234% of ip the highest slope (5.1×10^5^±6.2×10^4^) for the initial 5 min photon flux (Fig. 2b). This effect contributed directly to the faster biodistribution for iv injections (8.7 min less than ip) and the delay in the time-to-peak for sc (additional 4.4 min compared to ip) (Fig. 2c).

### 3. Anesthesia-dependent Photon Flux Changes

**Figure 3:**
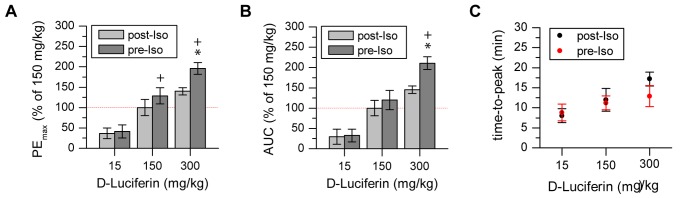
Luciferase inhibition by Isoflurane is avoided by substrate administration before anesthesia onset. a, b) Difference in PE_max_ and AUC under pre/post Isoflurane conditions becomes more pronounced with increasing substrate concentration. c) The order of application between Isoflurane anesthesia and substrate had no impact on the time-to-peak for 15, 150 and 300 mg/kg D-Luciferin. (* statistically significant difference with p≤0.05 to standard protocol 150 mg/kg post-Iso in post hoc comparison with Sidak correction;+statistically significant difference with p≤0.05 between pre and post condition in post hoc comparison with Sidak correction).

Based on previous reports describing the inhibitory effects of volatile anesthetics and the benefit of injection anesthetics like medetomidine [4], we sought to compare luciferase performance for common injection anesthetics: Pentobarbital and Ketamine/Xylazine. In a first experiment, DCX-Luc mice (n = 4) were injected with 15, 150 and 300 mg/kg D-Luciferin pre and post induction of Isoflurane anesthesia, in order to determine the influence of an anesthesia pre-inhibited enzyme. D-Luciferin injection pre Isoflurane anesthesia resulted in significantly increased PE_max_ (F(1, 3) = 51.585 p = 0.006) and AUC (F(1, 3) = 44.488 p = 0.007). By increasing the substrate concentration the maximum and total amount of photon flux difference for pre to post condition was significantly increased (300 mg/kg to 150 mg/kg pre Iso: PE_max_ p = 0.01, AUC p = 0.009) (Fig. 3a, b). The time-to-peak suggested a similar D-Luciferin concentration-dependent effect; this, however, did not reach the level of statistical significance (Fig. 3c).

**Figure 4:**
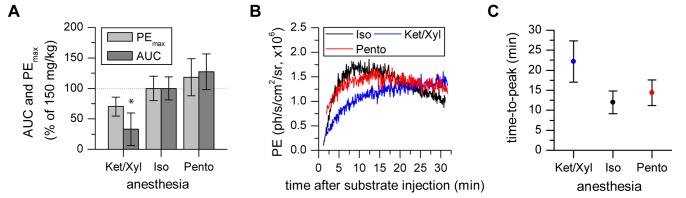
Photon flux is modulated by the type of anesthesia. a) PE_max_ and AUC were decreased under Ketamine/Xylazine conditions but not different for Pentobarbital compared to Isoflurane. b, c) Representative time activity curves showing the anesthesia dependent signal behavior leading to delayed time-to-peak for Ketamine/Xylazine, but no clear difference between Isoflurane and Pentobarbital anesthesia. (* statistically significant difference with p≤0.05 to standard protocol 150 mg/kg post-Iso in post hoc comparison with Sidak correction).

In a second experiment, brain specific BLI was compared for different anesthetics revealing an overall effect on PE_max_ (F(2, 6) = 5.935 p = 0.038) but no significant increase or decrease compared to Isoflurane anesthesia in post-hoc pairwise comparison (Ketamine/Xylazine: p = 0.058, Pento: p = 0.742). A significantly lower AUC value for Ketamine/Xylazine compared to Isoflurane (F(2, 6) = 22.738 p = 0.002; post-hoc p = 0.005) (Fig. 4) was accompanied by delayed time-to-peak values. No significant difference between the Isoflurane and Pentobarbital group was observed for time-to-peak.

### 4. Generation of Neural Stem Cells Expressing Luc2 and copGFP

**Figure 5:**
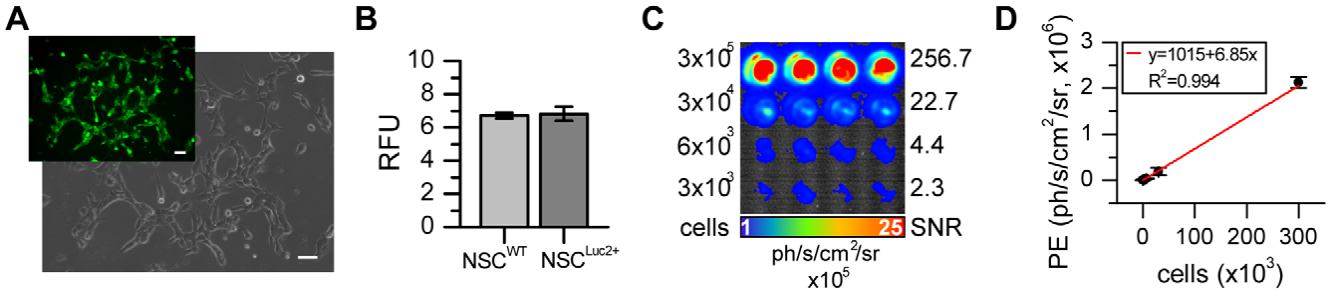
Characterization of Luc2-expressing NSCs. a) Efficient NSC transduction and selection process (by FACS and antibiotics) was confirmed by the homogeneous expression of the fluorescent reporter copGFP, which directly reflects Luc2 expression because of the T2A linker element (microscopic images 20× magnification, 50 µm scale bar). b) Reporter gene expression had no impact on cell viability, as confirmed with the PrestoBlue assay (data of 5 independent measurements presented as relative fluorescent units, RFU). c, d) Quantitative analysis of NSC*^Luc2+^* dilution series (1 min acquisition at 37°C with 30 µg/ml D-Luciferin) revealed a linear correlation between photon emission and cell number, as well as SNR in vitro.

The NSCs D3WT_N2Euro were stably transduced with the retroviral plasmid pBABE-Luc2-T2A-copGFP-Neo and sorted for copGFP/neomycin resistance to reveal a stable transgenic cell line without an effect on cell viability (Fig. 5). The NSC^Luc2+^ emit approx. 7 photons/s/cell under substrate excess (1 mM D-Luciferin) and show a linear correlation between cell number and photon emission under substrate excess.

### 5. Imaging Protocol-dependent in vivo Detection Limits

**Figure 6:**
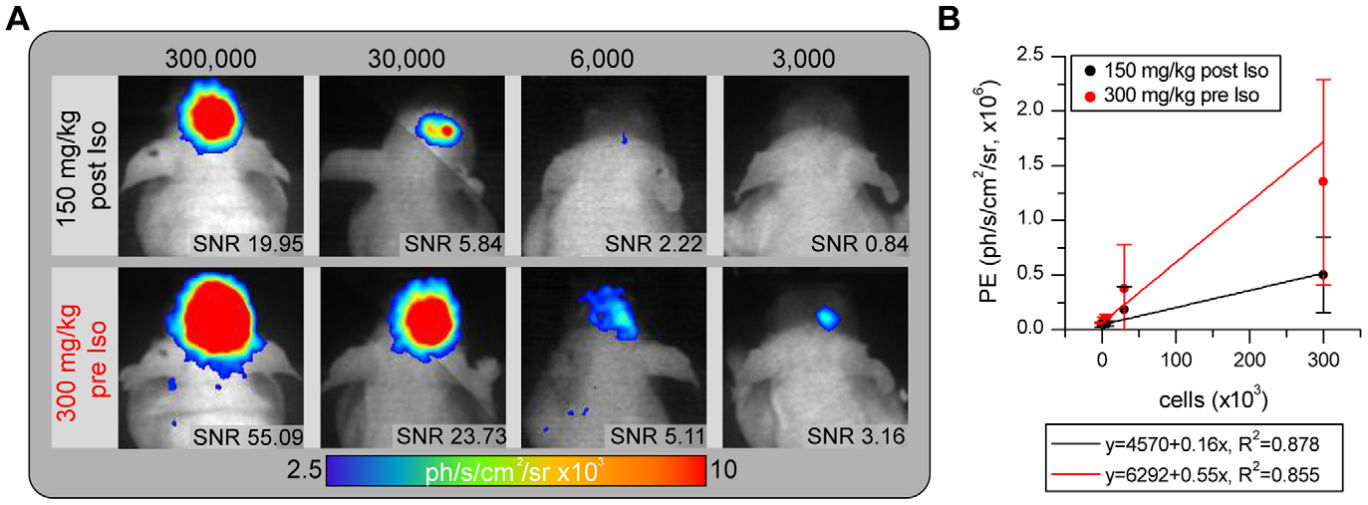
Photon emission is substantially increased by the modified BLI protocol. a) Representative images (equally scaled) for each cell number grafted into nude mice acquired with the standard protocol (upper row) and with the advanced protocol (lower row) reveal the objective sensitivity benefit, which is also represented in the quantitative SNR values. b) Correlation between photon emission and cell number revealed a linear relationship under in vivo conditions, with a steeper slope for the novel protocol, indicating the increased sensitivity.

BLI was performed 24 h after transplantation of 3,000, 6,000, 30,000 or 300,000 NSC^Luc2+^ into the striatum of nude mice (for each cell amount, n = 3), repeating the in vitro dilution series under in vivo conditions in order to determine detection limits. The mice were scanned with both protocols (150 mg/kg post Isoflurane and 300 mg/kg pre Isoflurane –6 h time separation) for 30 min. The BLI detection limit was calculated assuming a SNR≥3 for a reliably detectable signal. A representative image series with the corresponding SNR values is shown in Fig. 6a. This evaluation revealed the primary advantage of the new protocol, which, on average, leads to a 2.35-fold SNR increase. This sensitivity increase can be exploited for reliable detection of half the amount of cells visible using the standard protocol. The PE_max_ values for the grafts of various cell numbers, show a linear relationship with cell number (Fig. 6b).

## Discussion

Bioluminescence imaging is emerging as a valuable tool for tracking transplanted (stem) cells in various disease models [16]–[19]. However, a publication survey on 20 recent reports from 2008–2012, performed in preparation of the present study, revealed the lack of a consistent protocol for in vivo BLI, but with a strong tendency to 150 mg/kg D-Luciferin injected intraperitoneally which we have used as our reference “standard protocol”. This standard protocol has proved useful, primarily for peripheral applications, e.g. subcutaneous tumor cell transplants, where signal is mostly used and interpreted qualitatively. However, for a quantitative and sensitive bioluminescence imaging approach, this standard protocol does not necessarily meet the special requirements of brain imaging.

In this study, we evaluated the basic factors affecting the neuron-specific BL signal in transgenic DCX-Luc mice, including substrate concentration, route/timing of substrate administration and the type of anesthesia. The repetitive BLI measurements did not alter the BLI signal over time. As indicated by stability measurements (data not shown), repetition of the same protocol showed low variability of approx. 8%. The intrinsic inter-individual variability was 12±2%. An advanced protocol (300 mg/kg pre-Iso) was identified, which results in a 2-fold BL signal increase, while remaining user-friendly, fast applicable and cost-effective. We proved the advantages of the new protocol by boosting the sensitivity for a cell tracking approach in the mouse brain by a factor of 2 relative to the standard protocol, enabling the quantitative detection of 3,000 transplanted NSCs under the novel protocol conditions.

### 1. Concentration Dependent Increase in Photon Flux

The properties of the luciferase enzyme expressed in mammalian cells are well studied under in vitro conditions but missing for the in vivo situation – especially for brain specific BLI. We explored a dose-dependent BLI signal which followed an exponential increase. As was reported for 450 mg/kg D-Luciferin, in our experiments also 750 mg/kg did still not result in saturating photon emission levels. The exponential signal increase can be explained by a boost in substrate diffusion into the brain parenchyma exceeding a critical plasma level to facilitate blood brain-barrier penetration efficiently at 750 mg/kg. This is supported by the nearly 10-fold steeper slope, which most prominently represents the substrate inflow dependent photon emission in the first 5 min of acquisition.

Considering an in vitro firefly K_M_ of 1 mM or 0.3 mg/100 µl [20], this concentration should be reached by injection of 30 mg/kg D-Luciferin (assuming 2 ml total blood volume for a 20 g mouse). Nevertheless, by further increasing the substrate concentration, higher PE_max_ are achieved, which means that 30 mg/kg D-Luciferin is not the in vivo concentration at which the luciferase reaction rate is half of the maximum. Our results contradict previous assumptions, that the enzyme is already saturated at low substrate concentrations (5 µl of 100 mM) for subcutaneous applications in vivo [21]. However, under in vivo conditions substrate absorption and resorption is not 100% effective, meaning that not every injected molecule will reach a Luc-positive DCX neuron in the brain. Most importantly, Berger and colleagues calculated that only 5% of blood plasma D-Luciferin reach the brain [22], which explains the increasing luciferase activity at substrate concentrations which would already exceed K_M_ under in vitro conditions. At high substrate concentrations [21], the time-to-peak is significantly delayed. Under in vitro conditions, this would clearly be reflected in the conformational change of the luciferase enzyme, temporarily reducing the catalytic rate [23]. However, for the equivalent in vivo observation we rather suggest that high concentrations of D-Luciferin result in a prolonged concentration gradient between plasma and brain tissue facilitating a continued concentration increase at the target cells. A toxicity effect of the different D-Luciferin concentrations is not expected, as majority of studies have reported no toxic effect [24], [21], [25]–[27].

### 2. Influence of Injection Route

The advantage of D-Luciferin as an imaging substrate is its ability to distribute rapidly in the blood system, pass cell membranes and enters every organ (even through the placenta and blood brain-barrier) [21], [28], [29]. We validated the influence of the substrate injection route on the brain bioluminescence signal, by comparing sc, iv and ip injection of 150 mg/kg D-Luciferin and acquiring BLI in list mode for superior temporal resolution. According to reports about BLI of subcutaneously transplanted cells [5], [6], the substrate pharmacokinetics limits the bioluminescence signal. The absorption-independent iv injection leads to a 400% maximum photon emission in ¼ the time compared to the ip injection. In contrast, after ip injection the substrate is slowly absorbed into the vascular space, which limits bioavailability and finally reduces PE_max_ while prolonging time-to-peak [6], [30]. In contrast to a study of Inoue et al. the sc injection resulted in the lowest photon emission accompanied with a small slope and long time-to-peak value [5]. Although iv injection generates highest photon flux, we decided to use ip injection for the advanced protocol. The disadvantage of iv injections is the fast kinetics with lack of a plateau phase, which impedes reproducible measurements at the PE_max_ and induces additional error. The ip injection can be done with a high success rate [5], a shorter time (more mice can be measured the same time), repetitively without tissue irritation through tail vein catheterization and permits experimental flexibility by a prolonged steady-state BLI signal.

### 3. Anesthesia-dependent Photon Flux Changes

In vivo BLI of subcutaneously transplanted cells was reported to be strongly affected by the type of anesthesia [4]. Therefore, we investigated the effect of different anesthetics (Iso, Ket/Xyl, Pento) and the different anesthetic timing (D-Luciferin injection pre and post Iso) on brain-specific BLI. We observed a decrease in PE_max_ values for Ketamine/Xylazine anesthesia and a slight increase in PE_max_ for Pentobarbital anesthesia compared to our standard protocol using Isoflurane, however, this modulation did not reach a level of statistical significance. In vitro, Keyearts et al. found no direct inhibitory effect of Ketamine, Medetomidine (belonging to the same family of α2 adrenoreceptor agonists like Xylazine) or Pentobarbital [4]. Therefore, the modulation we observed in vivo may be the result of an altered hemodynamic situation, changing luciferase substrate distribution [31] and thereby indirectly influencing BLI signal intensity and kinetics. Nevertheless, anesthetics can have different effects on the blood system [32], [33]. In a comparison study, Isoflurane resulted in the smallest reduction of cardiac output, followed by Pentobarbital with medium reduction. The strongest impact on cardiac output is expected under Ketamine/Xylazine anesthesia, which induces hypotension, bradycardia and hypothermia [32], [34]. Although cerebral hemodynamics are autoregulated, the grading of the hemodynamic changes parallels our observation of delayed time-to-peak values for Pentobarbital and Ketamine/Xylazine anesthesia compared to Isoflurane.

The slight difference in PE_max_ between Pentobarbital and Isoflurane anesthesia may be due to the direct inhibitory effect of Isoflurane on the luciferase enzyme, which we studied by comparing pre vs post Isoflurane anesthesia. D-Luciferin injection before anesthesia consistently resulted in higher PE_max_/AUC compared to the standard protocol (substrate injection into the anaesthetized animal). Direct inhibition of the luciferase enzyme by Isoflurane was reported [35], [36]. It was previously suggested, that D-Luciferin inhibition by Isoflurane is a mixed style inhibition with competitive binding at the substrate binding site as well as a non-competitive binding changing the structure of the enzymatic pocket [37]. In line with previous reports, inhibition is partially reversible by higher substrate concentrations, which is due to an increased K_M_ value. Our data indicates that pre Isoflurane administration of D-Luciferin results in a better distribution of the substrate and hence in an increased availability of substrate in the brain compared to post Isoflurane injection. This may lead to an increased enzyme-substrate complex under pre-Iso condition, which is less affected by the inhibitory Isoflurane.

### 4. Imaging Protocol-dependent in vivo Detection Limits

We performed a quantitative comparison of the advanced vs. the standard imaging protocol applied to NSC^Luc2+^ transplanted into the mouse brain at different quantities. With this approach it was possible to study the detection limits of both protocols: 3,000 and 6,000 cells were not detectable with the standard protocol. Measuring these animals with the advanced protocol enables clear detection of even 3,000 transplanted NSCs (cf Fig. 6). To the best of our knowledge, such a small number of transplanted non-immortalized NSC has not been imaged in vivo before. We extracted SNR values for each graft size and determined that a SNR ≥3 can be used as a quantitative measure to reliably distinguish BLI signal from the noise. Visual comparison of all BL images (n = 12) confirmed that signals outside the cell graft exhibited SNR <3 and were identified as noise. Cell number estimation, as it was shown under in vitro conditions [38], seems to be possible in vivo providing that experimental conditions remain constant. High variability between animals receiving equal cell amounts was primarily induced by the transplantation procedure, which resulted in variations of cell number and transplantation depth, which strictly controls the percentage of transmitted bioluminescence light [39].

### Conclusion

Optical brain imaging remains challenging for quantitative and sensitive approaches like the tracking of small number of transplanted cells. Here, we empirically determined a novel and efficient protocol with ip injection of 300 mg/kg pre to Isoflurane anesthesia. This novel protocol turned out to be twice as sensitive as the conventionally applied “standard protocol”. Our quantitative analysis of the luciferase in vivo kinetics and the signal-to-noise ratio can serve as the basis of reliable detection limits for further studies.

## Supporting Information

Figure S1 **Background correction for ROI analysis of the DCX-Luc BLI data.** a) Representative overlay of BLI data and a photograph of a mouse during BLI acquisition. ROIs mark the area of data analysis on the head (upper circle) and on the back of the animal. b) The background data were subtracted from the brain data resulting in a data set corrected for the difference in Luc expression and D-Luciferin distribution among the animals. Each color represents one individual DCX-Luc mouse.
(TIF)
