# Supplementary figures and images for: Boosting Bioluminescence Neuroimaging: An Optimized Protocol for Brain Studies
Source: PLoS One. 2013 Feb 6;8(2):e55662. doi: 10.1371/journal.pone.0055662 (PMC3566035; doi:10.1371/journal.pone.0055662)

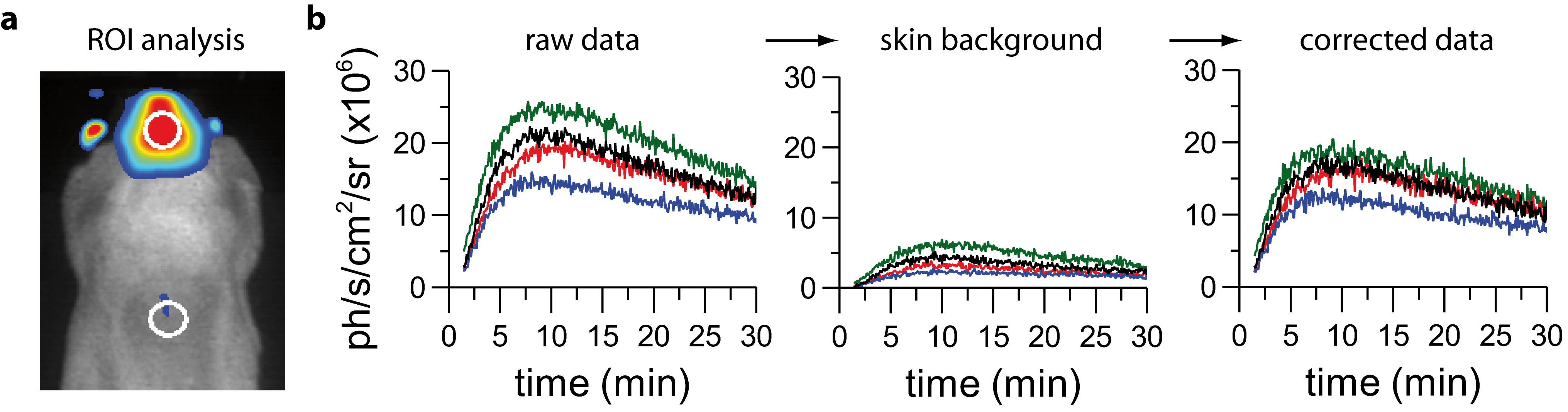

Supplement: Figure S1 — Background correction for ROI analysis of the DCX-Luc BLI data. a) Representative overlay of BLI data and a photograph of a mouse during BLI acquisition. ROIs mark the area of data analysis on the head (upper circle) and on the back of the animal. b) The background data were subtracted from the brain data resulting in a data set corrected for the difference in Luc expression and D-Luciferin distribution among the animals. Each color represents one individual DCX-Luc mouse. (TIF) [file pone.0055662.s001.tif]
